# Neurons in the dorsomedial medulla contribute to swallow pattern generation: Evidence of inspiratory activity during swallow

**DOI:** 10.1371/journal.pone.0199903

**Published:** 2018-07-19

**Authors:** Teresa Pitts, Ivan Poliacek, Melanie J. Rose, Mitchell D. Reed, Jillian A. Condrey, Hsiu-Wen Tsai, Guannan Zhou, Paul W. Davenport, Donald C. Bolser

**Affiliations:** 1 Department of Neurologic Surgery and Kentucky Spinal Cord Injury Research Center, College of Medicine, University of Louisville, Louisville, KY, United States of America; 2 Department of Physiological Sciences, College of Veterinary Medicine, University of Florida, Gainesville, FL, United States of America; 3 Comenius University in Bratislava, Jessenius Faculty of Medicine in Martin, Institute of Medical Biophysics, Martin, Slovak Republic; The Research Center of Neurobiology-Neurophysiology of Marseille, FRANCE

## Abstract

Active contraction of the diaphragm and other inspiratory pump muscles during swallow create a negative thoracic pressure to improve the movement of the bolus (food/liquid) into the esophagus. We tested the hypothesis that dorsomedial medullary inspiratory neurons, including the nucleus tractus solitarius (NTS, pre-motor to the phrenic) would be active during swallow induced by oral water infusion. We recorded neurons in the NTS and medial reticular formation in anesthetized spontaneously breathing cats, and induced swallow by injection of water into the oropharynx. Our results indicate that: *1)* a majority of inspiratory cells in the dorsomedial medulla are active during swallow, 2) expiratory neurons are present in the medial reticular formation (deeper to the NTS) in unparalyzed cats and a majority of these cells decreased firing frequency during swallow. Our findings suggest that the dorsomedial medulla is a source of inspiratory motor drive during swallow and that a novel population of breathing-modulated neurons that also are modulated during swallowing exist in the medial reticular formation in unparalyzed animals.

## Introduction

The “swallow-breath” (i.e. schluckatmung in German) was of great interest in the earliest experiments in deglutition [1, 2]. While there have been relatively few studies recently, Bosma [2] stated that the schluckatmung “has been studied extensively”, and references the work of several groups of European scientists. This issue was discussed by Marckwald [1] in Appendix I, with the ultimate conclusion that diaphragm movement is an active portion of swallowing produced by the swallow central pattern generator (CPG). Summarizing earlier work together with his studies in the rabbit he drew the following conclusions: a) the phrenic motor burst is approximately 200–300 ms after the burst of the mylohyoid; b) ablation of the phrenic nerve did not suppress all movement of the thorax during swallowing; c) central apnea (induced by barbiturate overdose) did not change the schluckatmung magnitude; and d) brainstem transection of the respiratory centers from the “swallow” centers abolished the schluckatmung.

During the 1960–70’s, with the use of esophageal manometry, the negative pressure during swallow was attributed to elongation of the esophagus termed “initial negative deflection” [3]. More recently, work by McConnel has termed it “hypopharyngeal suction pump” and reported it to be created by laryngeal elevation and the opening of the UES [4–9]. However, recordings during swallow in infants [10], adults [11], cats [12–15] and goats [16–18] demonstrate that electromyography (EMG) activity of inspiratory muscles occurs at the onset of swallow. More specifically, this diaphragmatic activity did not produce an inspiratory airflow, as measured with spirometry (i.e. airflow at the level of the mouth), but did produce a negative esophageal pressure deflection [11].

In general, it is understood that dorsal inspiratory neurons are active during swallow. Saito and colleagues [19] recorded from respiratory and non-respiratory neurons in the nucleus of the tractus solitarius (NTS) during fictive swallow in rats, and demonstrated that 19/53 inspiratory neurons were active during the hypoglossal burst. Additionally, Gestreau and colleagues [20] recorded from inspiratory neurons in the dorsal respiratory group of cats and reported that 28/33 neurons were active at the beginning of fictive swallow, however all displayed a peak discharge frequency lower than during breathing. Conversely, when we inspect examples from the presented figures the phrenic burst during swallow is very small compared to breathing-related activity. Of note, these studies used electrical stimulation of the superior laryngeal nerve to produce swallowing, which also produces profound suppression of phrenic motor neurons. Due to this, we believe that the phrenic activity during swallow (schluckatmung) has been classified as “unimportant” and not vital to appropriate behavioral execution. However, our working theory is that the functional contribution of the schluckatmung to swallow is to expand the thoracic cavity, creating a negative trans-diaphragmatic pressure, supporting bolus transition across the upper esophageal sphincter (UES) (i.e. similar to the movement of air during inspiration).

Bellingham and colleagues [21], during breathing, demonstrated that superior laryngeal nerve stimulation inhibits inspiratory output via a short latency (8ms) chlorine–dependent oligo-synaptic inhibition of phrenic motoneurons. Additionally, Sun, et al. [22] found that this inhibition is due to activation of expiratory-decrementing neurons in the rostral ventral respiratory group (Bӧtzinger Complex) via the NTS. In light of these studies, we hypothesized that the behavior of dorsal medullary respiratory neurons during swallowing induced by natural stimuli in the anesthetized cat model would be different than what was previously reported. Additionally we hypothesized that a higher number of dorsal inspiratory neurons would increase their activity during swallow relative to breathing. This information is vital to understand the regulation of the schluckatmung and its importance in the normal swallow motor pattern.

## Methods

Experiments were performed on six spontaneously breathing adult male cats. The protocol was approved by the University of Florida and University of Louisville Intuitional Animal Care and Use Committee (IACUC). The animals were initially anesthetized with sodium pentobarbital (Lundbeck, Inc., Deerfield, IL) (35 mg/kg i.v.); supplementary doses were given as needed (3–5 mg/kg i.v.). The right femoral artery and vein were cannulated to monitor blood pressure and administer i.v. fluids, respectively. Physiologic levels of end-tidal CO_2_ (4–4.5%), body temperature, and arterial blood gas composition were continually maintained and monitored.

EMGs were recorded using bipolar insulated fine wire electrodes according to the technique of Basmajian and Stecko [23]. Eight muscles were used to evaluate swallow occurrence: mylohyoid, geniohyoid, thyrohyoid, thyropharyngeus, thyroarytenoid, cricopharyngeus, parasternal, and costal diaphragm. These muscles span the actions during the pharyngeal phase of swallow: a) mylohyoid, geniohyoid and thyrohyoid for hyolaryngeal elevation; b) thyropharyngeus for inferior pharyngeal constrictor; c) cricopharyngeus for upper esophageal sphincter regulation; d) thyroarytenoid for laryngeal adduction; and e) parasternal and costal diaphragm for inspiratory activity [12, 13, 15, 24].

The digastric muscles were blunt dissected away from the surface of the mylohyoid and electrodes were placed in the left mylohyoid. A small horizontal incision was made at the rostral end of the right mylohyoid followed by an incision down the midline for approximately 5 mm to reveal the geniohyoid muscle. Electrodes were placed 1 cm from the caudal insertion of the geniohyoid muscle. The thyroarytenoid muscle electrodes were inserted through the cricothyroid window into the anterior portion of the vocal folds, which were visually inspected post-mortem. Minor rotation of the larynx and pharynx counterclockwise revealed the superior laryngeal nerve, which facilitated placement of the thyropharyngeus muscle electrodes. The thyropharyngeus is a fan shaped muscle with the smallest portion attached to the thyroid cartilage; electrodes were placed in the ventral, caudal portion of the muscle overlaying thyroid cartilage within 5 mm of the rostral insertion of the muscle. To place electrodes within the cricopharyngeus muscle, the larynx and pharynx were rotated counterclockwise to reveal the posterior aspect of the larynx. The edge of the cricoid cartilage was located by palpation and electrodes were placed in the cricopharyngeus muscle just cranial to the edge of this structure. Thyrohyoid muscle electrodes were inserted approximately 5 mm rostral to the attachment to the thyroid cartilage; those for the parasternal muscle were placed in the third intercostal space, just adjacent to the sternum, and the costal diaphragm EMGs were placed through the skin just under the xiphoid process. The positions of all electrodes were confirmed by visual inspection (following electrode placement and post-mortem) and by EMG activity patterns during breathing and swallow.

Swallowing was defined as a quiescence of cricopharyngeus (upper esophageal sphincter) activity with overlapping mylohyoid, geniohyoid, thyropharyngeus, thyrohyoid, thyroarytenoid, inspiratory muscle activity (parasternal/diaphragm). Swallow can be clearly differentiated from other behaviors (augmented breath, laryngeal elevation, cough, expiration reflex, and aspiration reflex) by this activity pattern [15, 25–28].

The animals were then placed prone in a stereotaxic frame and the dorsal surface of the medulla was exposed with removal of the cerebellum. The surface of the brain stem was covered with warm paraffin oil.

### Medullary recordings

**Fig 1:**
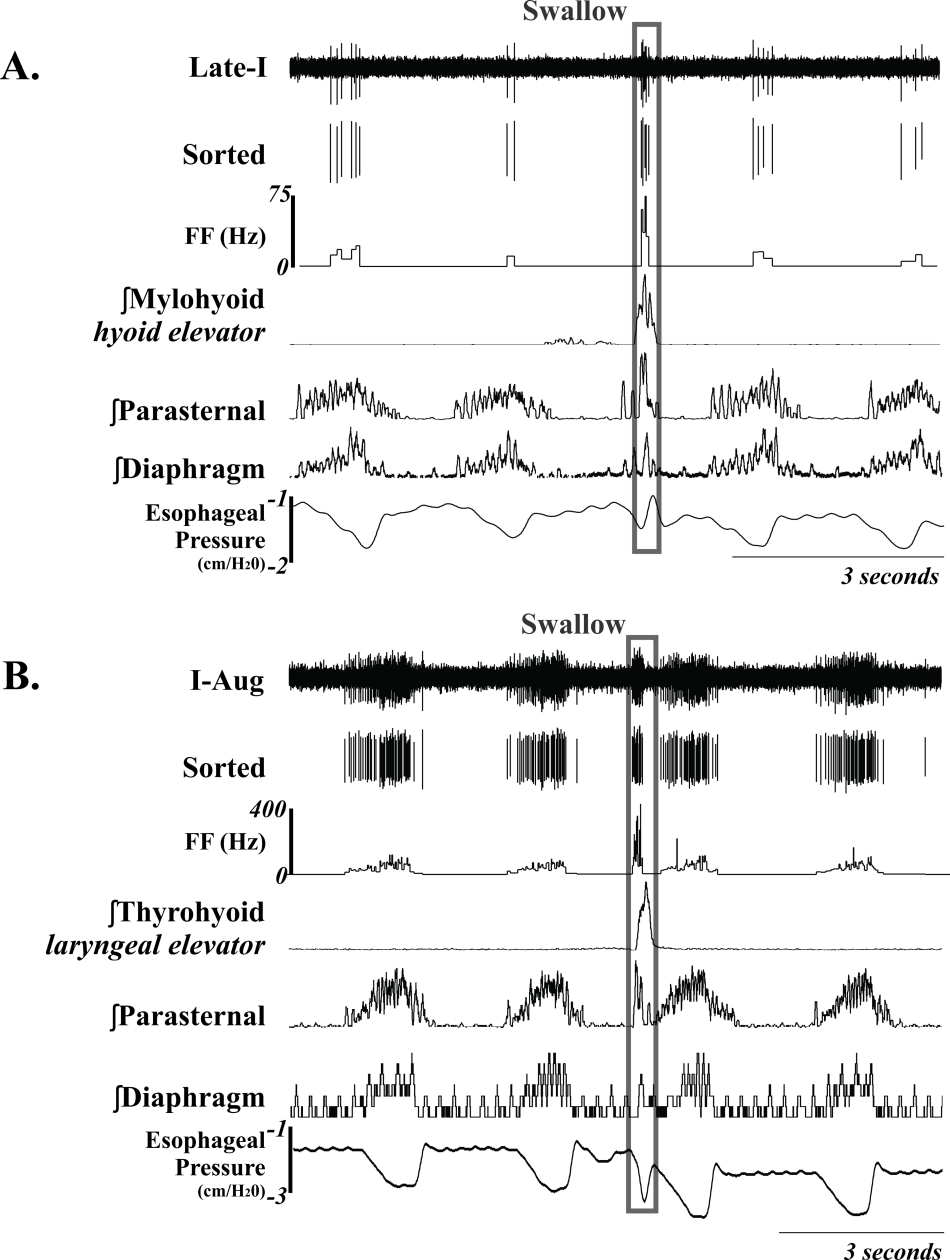
Example of two *NTS* Inspiratory neurons during breathing and swallow. Unsorted and sorted spike trains, and instantaneous firing frequency (FF) (Hz) are displayed; demonstrating an increase in FF during swallow. The swallow is outlined in the gray box. See Table 2 for anatomical location and Table 1 for neuron discharge pattern definitions. Recordings of EMG moving averages from the mylohyoid, thyrohyoid, parasternal, diaphragm and esophageal pressure are also shown.

**Fig 2:**
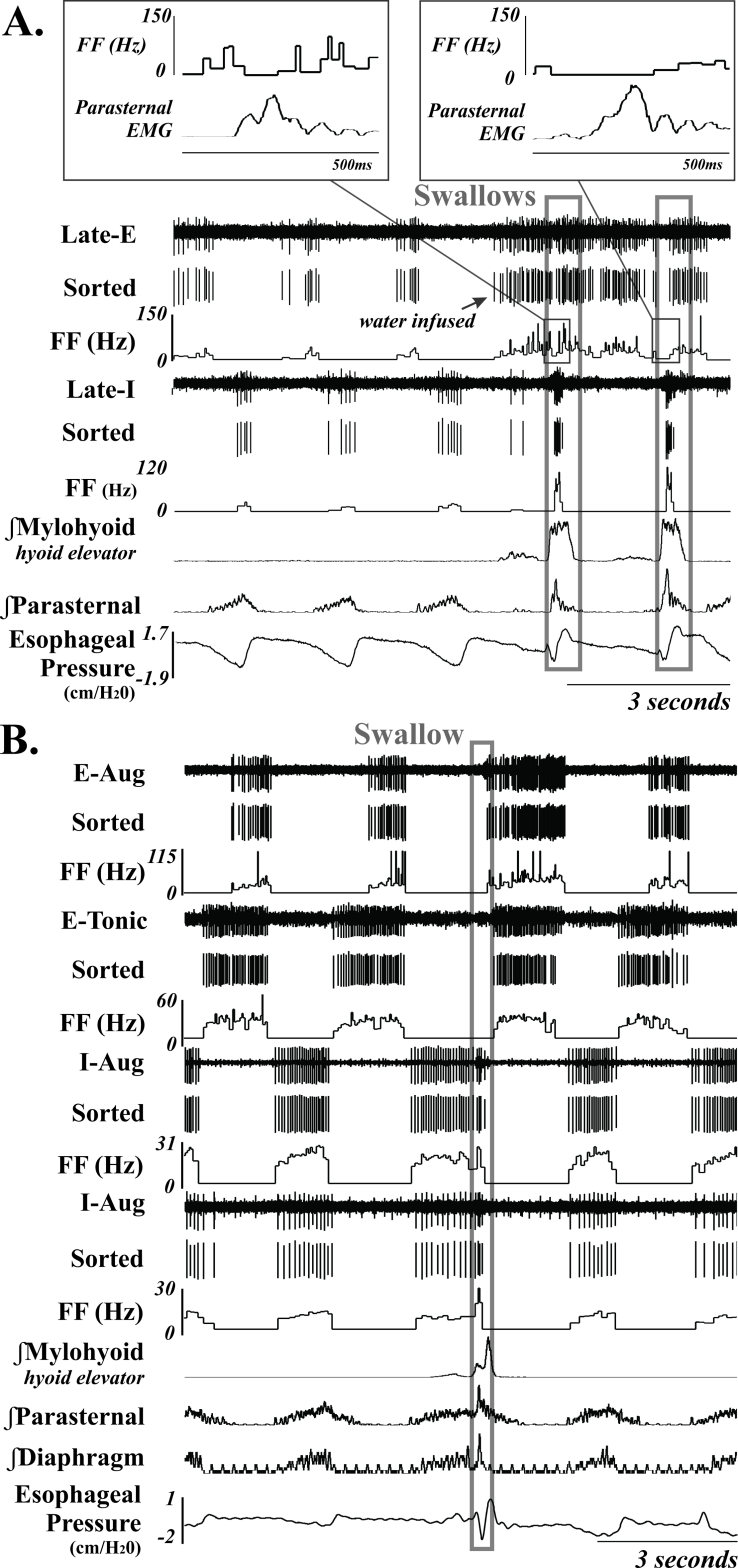
Example of 6 *MRF* neurons (3-I and 3-E) during breathing and swallow. Unsorted and sorted spike trains, and instantaneous firing frequency (FF) (Hz) are displayed; demonstrating an increase in FF during swallow for the two I neurons. **A** also demonstrates more complicated swallow-related changes in the Late-E neurons firing frequency, with the second example having a longer suppression duration. **B** demonstrates a representative example of suppression of an E neuron during swallow. They often fire across the entire E duration, except during the execution of swallow. The swallows are outlined in a gray box. See Table 2 for anatomical location and Table 1 for neuron discharge pattern definitions. Recordings of EMG moving averages from the mylohyoid, parasternal, diaphragm (B only) are also shown.

**Fig 3:**
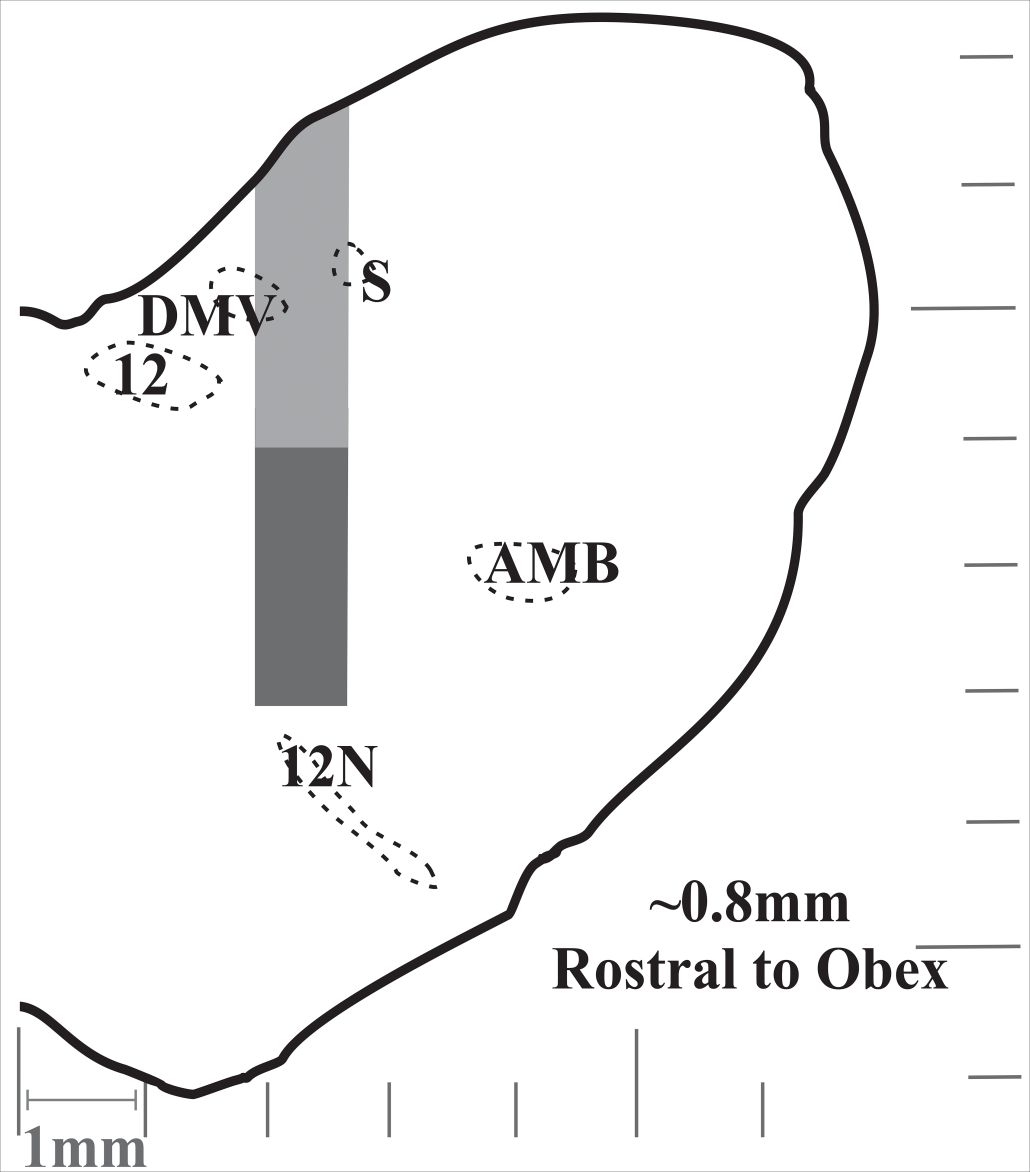
Display of recording location and depth at ~0.8mm rostral to obex. Light gray represents the NTS and dark gray the medial reticular formation (MRF). Acronyms: S (solitary track; DMV (dorsal motor nucleus of the vagus); 12 (hypoglossal nucleus); AMB (nucleus ambiguus); and 12N (hypoglossal nerve). Fig was modified from Berman [29].

Extracellular recordings of single respiratory neurons were made with tungsten microelectrodes (10–15 MΏ) arranged in an 8- or 16- channel array. The electrodes were arranged either linearly or within a rectangular grid with tips averaging 200–500 microns apart and individually advanced in micron steps, allowing isolation of signals from single neurons. The array was placed using stereotaxic coordinates derived from Berman [29]. Coordinates for the NTS (i.e. dorsal respiratory group) neurons were from the level of the obex to 2000 μm rostral, 1500–2400 μm lateral to the midline, and <2500 μm deep (Fig 1). Electrodes were routinely driven deeper than these coordinates into the medial reticular formation (MRF) (as deep as 4600 μm from the surface of the medulla) because the frequency of encountering breathing modulated neurons did not decrease with depth (Fig 2). The neurons were identified as inspiratory or expiratory by their discharge relationship to diaphragm/parasternal EMG during breathing. Recording locations are demonstrated in Fig 3.

### Elicitation of swallow

Following at least 5 breathing cycles swallow was elicited by infusion of 3ccs water into the oropharynx, via a one-inch long thin polyethylene catheter (diameter 0.5–1.0 mm), attached to a 6 ml syringe.

### Data processing and statistical analysis

Spike trains were analyzed off-line with Spike 2 software (Cambridge Electronic Design, United Kingdom). Templates were created from action potentials on each channel and subjected to cluster analysis to differentiate each template from noise and other simultaneously recorded neurons. Typically, each recorded channel consisted of well isolated action potentials that were easily differentiated from spikes attributed to other neurons that were more distant from the electrode. Figs 1 and 2 demonstrates signals from the electrodes, sorted spike times, and firing frequency (FF) in Hz. Moving averages of EMGs were integrated with a 200 ms time constant. Table 1 provides the firing pattern descriptors, acronyms and definitions. If the swallow maximum FF was greater than or less than 1 standard deviation (SD) away from the frequency during breathing the neuron was marked as “changed” with an indication of the direction [increasing (↑) and decreasing (↓)] (Table 2).

**Table 1:** Terms used, acronyms, and definitions for neuron discharge patterns.

Term	*Acronym*	Definition
Inspiratory	*I*	Maximum neuron firing rate during inspiration phase
Expiratory	*E*	Maximum neuron firing rate during expiration phase
Augmenting	*-aug*	Neuron whose firing rate increases throughout the phase
Decrementing	*-dec*	Neuron whose firing rate decreases throughout the phase
Tonic	*-tonic*	Neuron with no change in firing rate during the phase
Early	*early-*	Neuron with activity only at the beginning of the phase
Late	*late-*	Neuron with activity only at the end of the phase
Phase Span	*-phase span*	Neuron active across multi-phases of breathing

**Table 2:** Summary of neuron discharge patterns and changes in peak firing frequency (FF) during swallow for all recorded neurons. See Table 1 for neuron discharge pattern definitions.

	Total Cells	Increase^			Decrease^			No Change			Cell Active*			Not active**		
**NTS**	**18**	**7**	***39***	***%***	**7**	***39***	***%***	**4**	***22***	***%***	**16**	***89***	***%***	**2**	***11***	***%***
I-Aug	8	3	*38*	*%*	3	*38*	*%*	2	*25*	*%*	7	*88*	*%*	1	*13*	*%*
I-Dec	1	1	*100*	*%*						* *	1	*100*	*%*			
I-Tonic	4	2	*50*	*%*	2	*50*	*%*			* *	3	*75*	*%*	1	*25*	*%*
Late-I	2	1	*50*	*%*				1	*50*	*%*	2	*100*	*%*			
I-Phase Span	2			* *	2	*100*	*%*			* *	2	*100*	*%*			
			* *	* *		* *	* *		* *	* *		* *	* *		* *	* *
E-Tonic	1			* *				1	*100*	*%*	1	*100*	*%*			
				* *						* *			* *			
**MRF**	**68**	**21**	***31***	***%***	**32**	***47***	***%***	**14**	***21***	***%***	**57**	***84***	***%***	**10**	***15***	***%***
I-Aug	13	4	*31*	*%*	7	*54*	*%*	2	*15*	*%*	11	*85*	*%*	2	*15*	
I-Dec	7	5	*71*	*%*	1	*14*	*%*	1	*14*	*%*	6	*86*	*%*	1	*14*	
I-Tonic	16	9	*56*	*%*	3	*19*	*%*	4	*25*	*%*	16	*100*	*%*			
Early-I	2			* *				2	*100*	*%*	2	*100*	*%*			
Late-I	4	1	*25*	*%*	1	*25*	*%*	2	*50*	*%*	4	*100*	*%*			
I-Phase Span	3	1	*33*	*%*	2	*67*				* *	3	*100*	*%*			
			* *	* *		* *	* *		* *	* *		* *	* *		* *	* *
E-Aug	7			* *	5	*71*	*%*	1		* *	6	*86*	*%*	1	*14*	*%*
E-Dec	3	1	*33*	*%*	2	*67*	*%*			* *	1	*33*	*%*	2	*67*	*%*
E-Tonic	7			* *	7	*100*	*%*			* *	4	*57*	*%*	3	*43*	*%*
Early-E	1			* *	1	*100*	*%*			* *			* *	1	*100*	*%*
Late-E	4			* *	2	*50*	*%*	2	*50*	*%*	4	*100*	*%*			
E-Phase Span	1		* *	* *	1	*100*	*%*		* *	* *		* *	* *	1	*100*	*%*

*At least one action potential during swallow

** No action potentials present during swallow

^ ± 1 SD change in FF from breathing

Data is represented as mean ± SD. For statistical analysis Pearson chi-square test of independence was used to examine associations. A relationship was considered significant if the *p*-value was less than 0.05.

## Results

Eighty-six neurons were recorded from the dorsomedial medulla in the left brainstem. Infusion of water into the oropharynx resulted in a total of 297 swallows and 98% (291/297) occurred during the expiratory phase of breathing. Table 2 lists the recording site for each neuron with a peak-to-peak FF in Hz representation of its respiratory phase and peak FF change with swallow. Eighteen neurons were recorded within the region of the NTS (<2500μm), and 71 neurons within the MRF. Within the NTS 94% (17/18) were inspiratory (2 were phase spanning), 89% (16/18) were active during swallow, and 39% (7/18) increased in FF over that observed during breathing (Tables 2 and 3). The one E neuron in the NTS was active during swallow, but did not change its FF. Within the MRF 66% (45/68) of the recorded neurons were I (3 phase spanning), with the remaining 23 E (1 phase spanning). Of the MRF I cells 93% (42/45) were active during swallow, and 44% (20/45) increased their FF over that observed during breathing. While 65% (15/23) of the MRF E cells were active during swallow, 78% (18/23) decreased their FF during swallow (compared to breathing).

**Table 3:** Neurons recorded from the dorsomedial medulla described by neuron discharge identity; depth; coordinates for medial-lateral (ML); rostral-caudal (RC); the maximum peak-to-peak firing frequency (FF; in Hz) during breathing and swallow; and direction of change. Neurons are arranged by discharge identity (see Table 1 for descriptions) and the NTS/dorsal respiratory group neurons (< 2500 μm depth) are *italicized*. Neurons displayed in Figs are noted under the identity column.

M-L	R-C	Depth	Identity	Breathing			Swallow			Change*
**Inspiratory- Increase FF**										
*2281*	*1713*	*1332*	*I-Aug*	39	±	9	146	±	164	↑
*2581*	*900*	*1432*	*I-Aug*	43	±	6	65	±	38	↑
2000	350	2909	I-Aug	77	±	6	90	±	32	↑
2000	2688	4720	I-Aug	40	±	2	51	±	8	↑
*2000*	*688*	*672*	*I-Dec*	43	±	11	211	±	138	↑
2000	2300	3283	I-Dec	14	±	8	204	±	97	↑
2000	2300	3283	I-Dec	19	±	15	92	±	99	↑
2200	825	3488	I-Dec	83	±	14	106	±	194	↑
2000	725	3489	I-Dec	48	±	3	146	±	0	↑
2000	688	4505	I-Dec	47	±	10	181	±	139	↑
*2150*	*2138*	*2462*	*I-Tonic*	45	±	3	64	±	15	↑
*1900*	*725*	*2467*	*I-Tonic*	209	±	113	393	±	74	↑
2150	1875	2513	I-Tonic	33	±	1	218	±	84	↑
2100	1413	2922	I-Tonic	14	±	1	36	±	6	↑
2450	1413	2922	I-Tonic	13	±	1	37	±	1	↑
2255	750	3230	I-Tonic	18	±	2	266	±	233	↑
2000	2300	3283	I-Tonic	22	±	14	218	±	63	↑
2200	0	3347	I-Tonic	21	±	2	114	±	16	↑
2200	0	3347	I-Tonic	21	±	2	222	±	50	↑
2150	1875	3622	I-Tonic	278	±	109	583	±	273	↑
2000	688	4505	I-Tonic	3	±	2	314	±	112	↑
2255	375	3769	I-Tonic Fig 2B	84	±	5	103	±	50	↑
2481	1325	2568	Late-I	10	±	2	243	±	0	↑
2450	2588	3305	Late-I	12	±	7	190	±	215	↑
1880	1525	3914	Late-I	13	±	2	87	±	20	↑
2100	1825	4378	Late-I	10	±	1	36	±	15	↑
*2000*	*1238*	*608*	*Late-I* Fig 1A	22	±	8	82	±	10	↑
1880	1125	3926	Late-I Fig 2A	21	±	6	108	±	9	↑
**Inspiratory- No Change FF**										
2300	2588	3145	Early-I	294	±	252	276	±	255	—
2000	1913	4607	Early-I	46	±	14	45	±	10	—
*2268*	*375*	*1198*	*I-Aug*	298	±	121	173	±	77	—
*2250*	*325*	*2062*	*I-Aug*	143	±	48	160	±	46	—
2150	1950	2807	I-Aug	152	±	220	96	±	64	—
2000	2300	4406	I-Aug	736	±	246	713	±	446	—
*2150*	*775*	*2450*	*I-Aug* Fig 1B	77	±	24	90	±	44	—
2000	725	3489	I-Dec	503	±	185	478	±	268	—
2255	375	2583	I-Tonic	576	±	389	722	±	347	—
2150	1950	2807	I-Tonic	83	±	21	92	±	126	—
2100	1950	2946	I-Tonic	346	±	256	110	±	66	—
2250	1113	3236	I-Tonic	23	±	1	1	±	0	—
2250	2138	4130	I-Tonic	11	±	0	8	±	11	—
*2181*	*900*	*2260*	*Late-I*	11	±	3	12	±	21	—
2150	2700	3681	Late-I	22	±	19	34	±	0	—
1880	375	3920	Late-I	51	±	19	38	±	54	—
**Inspiratory- Decrease FF**										
*2245*	*0*	*1943*	*I-Aug*	247	±	208	0	±	0	↓
*2250*	*325*	*2159*	*I-Aug*	111	±	16	80	±	22	↓
*2245*	*375*	*2258*	*I-Aug*	342	±	19	269	±	124	↓
2000	2263	2579	I-Aug	35	±	9	0	±	0	↓
2400	2588	3305	I-Aug	41	±	12	1	±	0	↓
2481	1713	3375	I-Aug	51	±	3	0	±	0	↓
2275	750	3944	I-Aug	67	±	10	0	±	0	↓
1880	1950	4120	I-Aug	38	±	3	18	±	25	↓
2300	2213	4552	I-Aug	251	±	92	83	±	63	↓
2255	1413	4554	I-Aug	33	±	1	24	±	1	↓
2200	825	3043	I-Dec	273	±	49	0	±	0	↓
*2150*	*0*	*1612*	*I-Tonic*	91	±	14	57	±	21	↓
*2245*	*0*	*1943*	*I-Tonic*	407	±	386	0	±	0	↓
*2250*	*325*	*2062*	*I-Tonic*	43	±	10	24	±	19	↓
*2150*	*325*	*2349*	*I-Tonic*	805	±	180	297	±	212	↓
2100	1950	2946	I-Tonic	600	±	424	29	±	23	↓
2181	1713	3221	I-Tonic	54	±	9	16	±	19	↓
1880	1950	3639	I-Tonic	175	±	34	11	±	6	↓
2000	1550	3766	I-Tonic	46	±	2	25	±	21	↓
2250	2138	4130	I-Tonic	11	±	1	8	±	11	↓
2100	2700	3078	Late-I	34	±	6	5	±	4	↓
**Expiratory- Increase FF**										
2000	725	4145	E-Aug	109	±	0	126	±	4	↑
1880	1125	3662	E-Dec	76	±	4	87	±	25	↑
2000	2300	4406	E-Tonic	34	±	3	219	±	315	↑
**Expiratory- No Change FF**										
2255	750	3307	E-Late Fig 2A	51	±	14	60	±	85	—
*2200*	*2438*	*803*	*E-Tonic*	43	±	16	56	±	12	—
1900	1913	4269	Late-E	61	±	18	71	±	25	—
**Expiratory- Decrease FF**										
1880	1525	3881	Early-E	10	±	3	0	±	0	↓
2250	1875	3438	E-Aug	41	±	6	0	±	0	↓
2000	1550	3570	E-Aug	108	±	64	4	±	0	↓
1900	2688	3662	E-Aug	411	±	846	9	±	13	↓
1900	1913	3699	E-Aug	61	±	10	32	±	49	↓
2000	2688	4137	E-Aug	36	±	2	18	±	22	↓
2255	1200	3532	E-Dec	22	±	8	0	±	0	↓
2255	1413	3621	E-Dec Fig 2B	23	±	1	0	±	0	↓
2000	750	3427	E-Tonic	18	±	4	12	±	9	↓
2481	2438	3525	E-Tonic	343	±	477	31	±	0	↓
2000	1125	3536	E-Tonic	31	±	4	0	±	0	↓
1880	1950	3639	E-Tonic	7	±	1	0	±	0	↓
1880	2300	4053	E-Tonic	30	±	4	0	±	0	↓
2000	1713	4084	E-Tonic	19	±	8	1	±	0	↓
2000	2300	4406	E-Tonic	43	±	2	0	±	0	↓
1900	2688	4572	E-Tonic	33	±	19	4	±	8	↓
1900	2688	3662	Late-E	23	±	3	4	±	8	↓
1900	2300	4246	Late-E	24	±	6	4	±	10	↓

NTS neurons are italicized (depth <2500)

*Greater than or less than 1 SD away from the mean FF during breathing.

Inspiratory neurons that increased their firing frequency did so by an average of 820 ± 2115%. Inspiratory neurons who decreased their firing frequency did so by an average of 69 ± 30%, in contrast, expiratory neurons decreased their firing frequency by an average of 90 ± 16%.

A Pearson chi square test was performed to examine the relationship between respiratory phase and increase in firing frequency during swallow [X^2^ (1, *n* = 86) = 13.5, *p* < 0.001]; indicating inspiratory neurons in the dorsal medulla are more likely to increase firing frequency during swallow compared to expiratory neurons. Additionally, to examine the relationship between respiratory phase and the presence of neurons active during swallow [X^2^ (1, *n* = 86) = 8.49, *p* = 0.004]; indicating inspiratory neurons in the dorsal medulla are more likely to be active during swallow compared to expiratory neurons.

See S1 Text. NC3Rs ARRIVE Guidelines Checklist in Supporting Information for more information.

## Discussion

This is the first study to report swallow-related firing frequency increases in inspiratory neurons in the dorsal medulla during swallow. The major findings of this study were that: of the recorded neurons in the dorsal medulla 73% of I neurons were active during swallow with 37% increasing in FF, and 67% of E neurons were active with 75% decreasing their FF. Additionally, we characterized a population of I and E neurons in the MRF during breathing and swallow which appears to be “anatomically” continuous with the NTS (Fig 3). Together this data expands our understanding of the overlapping regulation of breathing and swallow. Additionally, the increase in NTS I activity supports our hypothesis that the swallow pattern generator activates pre-motor neurons in the DRG for diaphragm recruitment during swallow (i.e. schluckatmung).

In freely breathing cats, I neurons in the region of the NTS that increased their FF during swallow relative to breathing did so by an average of 820%. The ventrolateral NTS contains a population of I neurons that has been termed the Dorsal Respiratory Group (DRG). Most of these I cells are bulbospinal and pre-motor to phrenic motoneurons [30–32]. Phrenic motoneurons innervate the diaphragm, and Figs 1 and 2 have examples of swallow demonstrating schluckatmung activity on parasternal and costal diaphragm EMG’s. However, FF of pre-motor neurons significantly contributes to motor drive, and our results demonstrate that it is possible for the pre-motor drive to inspiratory muscles to be higher during swallow when compared to breathing. Previously published recordings in decerebrate, decerebellate, paralyzed, ventilated cats, and using superior laryngeal nerve stimulation (SLN) to initiate swallow reported no I neurons increasing their FF during swallow [19, 20]. Of importance, neither of these papers discussed this I neuron activity relating to the schluckatmung as being important to the production of swallow. The lack of studies on I neuron activity during swallow is likely explained by two issues: a) the schluckatmung has not been universally accepted as a key feature in the swallow motor pattern; and b) the reported weak/reduced FF of I neurons during swallow lead to a theory that this activity is not “robust” enough to be important.

### History of the research on inspiratory activity during swallow

Rosenthal [33] was the first to report on the diaphragm contractions during stimulation of the SLN, however Arloing [34] was the first to provide substantial evidence that these contractions are an active part of the swallow pattern, creating the negative deflection during swallow. It was hypothesized that the diaphragm activity produced a negative deflection in the thoracic cavity creating suction to pull the bolus (food/liquid) into the esophagus. Our results support the hypothesis that NTS I neurons contribute to I motor drive during swallow to generate this negative intrathoracic pressure. Additionally, we only recorded one E neuron with stereotaxic coordinates within the NTS and this finding is consistent with other reports i.e. Berger [30] and Wallois et al. [35].

McConnel and colleagues [4, 5, 36–40] postulated that the tongue force and negative esophageal deflection had the largest impact on movement of the bolus through the esophagus. It was further hypothesized that the “initial negative deflection” during the pharyngeal phase of swallow is achieved by the elevation of the larynx and opening of the upper esophageal sphincter (UES). We have strongly proposed that this function is by inspiratory muscle activity acting as an aspiration generating force (negative pressure), and it a vital part of the swallow motor pattern [12–15, 24].

### Medial reticular formation (MRF)

We also recorded from a large population of respiratory neurons deeper than the NTS, in a region consistent with the medial reticular formation. One-third of this population discharged in phase with expiration. The occurrence of respiratory modulation of medial reticular formation neurons has been previously described in a series of papers from Lambertz, Langhorst, and Schultz in freely breathing dogs [41–44]. They also made extracellular recordings from a larger area (both rostral and caudal to obex) and reported that 30% had maximum activity during inspiration and 25% during expiration [41]. We propose that our experimental preparation (unparalyzed) increased the probability of encountering these neurons as paralyzed animals have historically been employed to investigate the locations of breathing-modulated neurons in the medulla.

The extent to which these neurons receive excitatory input from the central pattern generator for breathing in unknown. Neurons found in the MRF often receive feedback from somatic afferents via projections from spinoreticular neurons [45–47]. The source of respiratory drive to this population is less important than their function. Multiple anatomical studies have shown that neurons in this region of the medulla are members of pathways that are relevant to licking and chewing for example [48–50]. Further, some neurons in the medial reticular formation are pre-hypoglossal [51]. Our results show that some I neurons in this region increase their discharge rate during the schluckatmung, suggesting that they participate in swallow generation. In contrast to the activation of inspiratory cells, 83% of the expiratory cells had a decrease in firing frequency with 8/23 having no action potentials during swallow. As abdominal EMG suppression occurs during swallow [24], it is possible these expiratory neurons provide motor drive to spinal expiratory motor pathways, in addition to the caudal ventral respiratory column.

### Critique of methods

This study had several limitations. The first is an inflation test was not performed to separate NTS I cells that are pre-motor to the diaphragm versus pump cells (i.e. responding to lung inflation). Secondarily, there are known effects of anesthesia on respiratory drive (i.e. reduction of central excitability and increase in CO_2_ tolerance) and possible suppression of the schluckatmung. However, we felt it was important to test the earlier work described by Marckwald [1], and determine if the experimental preparation may bias appreciation of the schluckatmung as an important part of the swallow pattern.

## Conclusion

Inspiratory neurons in the dorsomedial medulla (including the NTS) contribute to the production of the of schluckatmung by increasing motor drive to inspiratory muscles. The schluckatmung is a reliable and robust portion of the swallow pattern in anesthetized animals. A novel population of expiratory neurons was identified in the medial reticular formation, deep to the NTS, that decreases activity during swallow, consistent with depression of expiratory motor drive that has been identified during this behavior. In conclusion, these results support the existence of an expanded neural network for the generation of swallowing that includes the medial medulla deep to the NTS.

## Supporting information

S1 Text NC3Rs ARRIVE guidelines checklist. (PDF)
